# Implications of COVID-19 pandemic and response approaches in Uganda: Stakeholder perspectives

**DOI:** 10.4102/jphia.v16i1.697

**Published:** 2025-03-05

**Authors:** Moses Ocan, Josephine Bayigga, Adelline Twimukye, Hellen Nansiiro, Daphine Sanger, Mordecai Tayebwa, Boniconsilli Tusiime, Maureen Katusiime, Daniel Kyabayinze, Benon Kwesiga, Henry K. Bosa, Leah Mbabazi, Tonny Muwonge, Brenda N. Simbwa, Francis Kakooza, Mosoka P. Fallah, Alex R. Ario

**Affiliations:** 1 Department of Pharmacology and Therapeutics, College of Health Sciences, Makerere University, Kampala, Uganda; 2 Infectious Diseases Institute, College of Health Sciences, Makerere University, Kampala, Uganda; 3 Department of Grants, College of Health Sciences, Makerere University, Kampala, Uganda; 4 Department of Health Policy and Management, College of Health Sciences, Makerere University, Kampala, Uganda; 5 Department of Public Health, Faculty of Disease Prevention, Ministry of Health, Kampala, Uganda; 6 Department of Clinical Services, Clinical Faculty, Ministry of Health, Kampala, Uganda; 7 Clinical Department, Faculty of Disease Prevention, Ministry of Health, Kampala, Uganda; 8 Department of Epidemiology, Uganda National Institute of Public Health, Ministry of Health, Kampala, Uganda; 9 Department of Epidemiology, Faculty of Disease Prevention, Africa Centers of Disease Control and Prevention, Addis Ababa, Ethiopia

**Keywords:** COVID-19, key stakeholder, pandemic, Uganda, implication

## Abstract

**Background:**

The coronavirus disease 2019 (COVID-19) pandemic significantly impacted health systems worldwide.

**Aim:**

This study explored the effects of the COVID-19 cascade on health programmes in Uganda.

**Setting:**

This study conducted in-depth interviews with key informants involved in Uganda’s national COVID-19 response.

**Methods:**

A cross-sectional exploratory study using qualitative approaches was carried out. A purposive sample of 30 key informants from the Ministry of Health (MoH) and implementing partners were interviewed (May 2023 – June 2023). Interviews were audio recorded and analysed using inductive thematic analysis with NVivo 14 software.

**Results:**

Four themes emerged: (1) approaches and opportunities for successful COVID-19 response, (2) negative impacts of the pandemic on health services, (3) barriers to implementing response strategies and (4) suggestions for preparedness for future epidemics.

**Conclusion:**

While the pandemic disrupted health programmes and access to care, it also revealed opportunities to strengthen healthcare delivery. Strengthening the dedicated Ministry of Health department for epidemic preparedness and response is recommended.

**Contribution:**

This study identifies areas for improvement in Uganda’s health system exposed by the COVID-19 pandemic. It informs public health preparedness efforts in Uganda and other African countries, aligning with the Journal’s focus on strengthening health systems in Africa.

## Introduction

The World Health Organization (WHO) declared coronavirus disease 2019 (COVID-19) as a Public Health Emergency of International Concern (PHEIC) on 11 March 2020, owing to a 13-fold increase in cases outside China and a three-fold increase in the number of countries affected by the pandemic. Over 118 000 cases had been registered, and 4291 people had lost their lives in 114 countries by then.^1^ Uganda then instituted measures to prevent the pandemic from affecting its population and identify cases through surveillance systems and mandatory testing and screening among travellers coming from countries categorised as high-risk coupled with a 14-day compulsory quarantine of this group.^2^

Despite efforts to control COVID-19, Uganda experienced its first COVID-19 case on 22 March 2020.^3^ The government responded with more stringent measures to prevent further spread of COVID-19. These included restricting public gatherings, international travel bans, importing essential goods and introducing a curfew.^2^ The pandemic affected almost all sectors of the economy, including health, trade, tourism, transport and agriculture, which are often associated with introducing lockdown measures by the government. From an economic perspective, restricted movements implied labour shortage, cutting production and supply and consumption challenges.^4,5,6,7,8^

In several African countries, healthcare workers reported significant disruptions to the healthcare system during the COVID-19 pandemic.^9^ Key challenges included widespread fear among healthcare workers of contracting the virus, leading to increased absenteeism. This was exacerbated by COVID-19 prevention measures, such as transport restrictions and social distancing, which further reduced staff availability. Healthcare workers who remained on duty were faced with a high workload compounded by a pervasive lack of updated knowledge on COVID-19 because of limited training and fewer opportunities for meetings on new health guidelines. This hindered their ability to respond to questions on the pandemic effectively. The pandemic also led to the closure of some non-essential health services, such as circumcisions, dental care and pap smears. Furthermore, supply chain disruptions caused stockouts of essential medicines, with some drugs being reserved solely for emergency care.^9,10^

The onset of the pandemic in Uganda followed a series of approximately eight episodes of zoonotic diseases in recent years, spanning from 2009 to 2014 and resurging in 2017 and 2018.^11^ In addition to grappling with these challenges, the country successfully navigated through six instances of Ebola outbreaks.^12^ Furthermore, over the years, Uganda has had multiple outbreaks, including malaria, anthrax, cholera, Rift Valley fever, Crimean Congo haemorrhagic fever and yellow fever, among others.^13^ All these epidemics have had various impacts on the health system. Henceforth, COVID-19 compounded the effect on the country’s implementation of health programmes despite the health system’s resilience.

The narrative of Uganda’s response to the pandemic was commended, contrasting favourably with its East African counterparts, Tanzania, Kenya and other low- and middle-income countries.^14,15^ This commendation stems from the system’s remarkable capability to mitigate COVID-19 effects. Drawing insights from Uganda’s rich experience in handling various outbreaks, this study dissected the management of the pandemic and assessed the implications of diverse responses to health programmes. Such an exploration promises valuable lessons for future pandemics, shedding light on Uganda’s ability to navigate health crises effectively.

## Research methods and design

### Study design

This was a cross-sectional exploratory study conducted using qualitative methods. The phenomenological approach was utilised to explore experiences and events during COVID-19 and their effect on healthcare programmes in Kampala, Uganda. Data were collected through key informant interviews (KIIs). Phenomenology theory was employed to generate concepts based on stakeholders’ experiences and perceptions regarding the impact of COVID-19, drawing on their expert perspectives.^16,17^

### Setting

Uganda’s healthcare system operates through decentralised services managed by district health teams across 112 districts, overseen by the central Ministry of Health (MoH). Each district, led by a District Health Officer and a team of health managers, manages decision-making for health services, including budgeting and resource allocation. The district health teams coordinate with other departments, such as education and agriculture, under the authority of the Chief Administrative Officer and Local Council V chairperson. Health services are provided through a network of facilities, including General Hospitals, Health Centre IVs, IIIs and IIs, with Village Health Teams (VHTs) offering community-level care. Both public and private not-for-profit sectors play significant roles, with healthcare service provision by a range of professionals, including physicians, nurses, midwives and technicians at various levels of care.

### Study population and sampling strategy

Key informant interviews were conducted with MoH officials and implementing and development partners including the Centers for Disease Control and Prevention (CDC), the United States Agency for International Development (USAID) and WHO at both from strategic planning and implementation levels of the health system. Participants were purposively selected based on their affiliation with MoH programmes, their minimum of 1 year of service in their respective roles, their active involvement in addressing the challenges posed by COVID-19 in Uganda, and their significant contributions to implementing or influencing COVID-19 and essential health programmes within the country. This criterion ensured that participants had sufficient knowledge of both the previous and current state of the pandemic, as well as the programmes they represented, allowing for informed insights into the ongoing response and challenges.

### Sample size and sampling

The sample size was determined by the representation of essential services defined by managers of essential health programmes and ministry technical staff. The inclusion criteria were a willingness to share information and to provide informed consent. A total of 30 respondents, including 15 MoH technical staff and 15 implementing and development partners who were involved in the national COVID-19 response, were purposively selected to gain an in-depth understanding of how COVID-19 evolved, was managed and had implications for healthcare programmes. A list of selected participants with their phone contacts was given to the research assistants who contacted the respondents.

### Data collection

Selected participants were given a phone call by the interviewers, information about the study was shared, and a convenient appointment and meeting place. All interviews were conducted at the offices of the respondents privately. All contacted participants accepted to participate in the study. The interview guide used in our study was designed by the study team to ensure open-ended questions and probes on the evolution of COVID-19, approaches to management, effects on health services and possible recommendations for a response to future pandemics. This approach helped to ensure that the interviews were structured in a way that would yield relevant and insightful information for our research. Some of the questions in the interview guide centred around the evolution of COVID-19 in Uganda, experiences during the COVID-19 pandemic, including service delivery, the impact of COVID-19 on the quality of health services and utilisation, perceptions on the impact of the healthcare system and future recommendations for addressing these challenges (Online Appendix 1). The study team conducted rigorous pretesting through meetings and dry runs to ensure that the tool effectively addressed the study objectives. This thorough testing process was crucial in refining the tool and ensuring it accurately captured the necessary information to meet the research goals. Three female study team members (D.S., N.H. and E.R.) with a background in social sciences (Bachelor of Arts [BA]) and public health (Bachelor of Public Health [BPPH]) respectfully and good clinical practice training were further trained on the collection of study data using the study interview guide, including note-taking. The interviewers were employed as research assistants and research coordinators (N.H.) at the time of the study. They have qualitative research experience of over 7 years on average with Makerere College of Health Sciences. Interviewers had no established relationship with respondents prior to the interviews. Written informed consent was obtained from each respondent before their participation in the study. All interviews were conducted in English, and each key informant participated in a single round of interviews. The interviews were audio recorded and supported by written notes, which served as valuable references for responses obtained and as backups to the recordings. On average, they took 45 minutes. Data saturation was reached at data collection from the purposefully selected respondents, we also observed thematic saturation during analysis.

### Data analysis

Audio recordings were transcribed verbatim, and a quality control process involving proofreading and relistening to the audio was employed to guarantee the accuracy and completeness of the transcripts. The qualitative analysis involved three independent coders (A.T., J.B. and N.H.). Eight transcripts from equally representative key informant categories were selected for open coding to develop a coding framework through an inductive content analysis approach. Each coder reviewed the transcripts and identified key concepts defined as codes. These were crosschecked among the three coders for a consensus and to increase the reliability of the coding process. Revised codes were organised into categories, and emerging themes were identified. Co-investigators actively participated in the review of these categories and themes, providing additional layers of validation and consensus-building. The conceded coding framework was then entered into NVivo 14 software. Transcripts were subsequently imported into NVivo software, facilitating open coding, data management and structured inductive analysis. We identified codes, themes and patterns in the interview texts that addressed the study objective. Our findings are presented with illustrative quotations selected from the most frequent codes of each emergent theme. Our approach to data analysis enhances the reliability and credibility of our findings, providing an in-depth understanding of the implications of the COVID-19 pandemic on health programmes in Uganda.

### Ethical considerations

The study protocol received ethical review and approval from the Makerere University School of Biomedical Sciences Research Ethics Committee (reference no.: SBS-2022-261) and clearance from the Uganda National Council for Science and Technology (reference no.: HS2664ES). Administrative clearance was obtained from the Ministry of Health and Hospital Administration. Written informed consent was obtained from respondents before the interviews were conducted.

## Results

### Participants’ characteristics

We interviewed a total of 30 participants (15 MoH technical staff and 15 development and implementing partners in health). On average, the respondents had worked for 5 years. The majority, 73.3% (*n* = 22/30), were males (Table 1).

**TABLE 1:** Summary of participants’ characteristics.

Category	Representatives (*n*)	%
**Participants’ categories (*N* = 30)**		
Development partners	8	26.7
Implementing partners	6	20.0
Ministry of Health	16	53.3
**Gender (*N* = 30)**		
Male	22	73.3
Female	8	26.7
**Programmes represented (*N* = 30 [100%])**		
Emergency medical services	4	13.3
Laboratory services	3	10.0
Epidemiology	2	6.7
Incident management	10	33.3
Logistics	3	10.0
Taskforce	6	20.0
Vaccination	2	6.7

Note: Average years of experience in the field – 5 years.

The results were organised into four themes: (1) approaches and opportunities for successful COVID-19 response, care and management; (2) negative effects of the COVID-19 pandemic on health service delivery in Uganda; (3) barriers to implementing COVID-19 response strategies and (4) suggestions for preparedness for future epidemics (Table 2).

**TABLE 2:** Study’s themes.

Theme	Codes
1. Approaches and opportunities for successful COVID-19 response, care and management	1.1. Well-coordinated COVID-19 response 1.2. Health facility response 1.3. Integration 1.4. Communication strategy 1.5. Risk communication and digitisation 1.6. Building on existing capacity and experience
2. Negative effects of the COVID-19 pandemic on health service delivery	2.1. Disruption of routine services 2.2. Pressure on health resources including an increased workload
3. Barriers to implementation of COVID-19 response strategies	3.1. Government mistrust 3.2. Dynamic nature of the pandemic 3.3. Violation of protocols 3.4. Inadequate funds
4. Suggestions for future preparedness during pandemics	4.1. Focus on the continuity of health services amid pandemics 4.2. To improve public awareness 4.3. To improve surveillance 4.4. To improve coordination and leadership 4.5. Investment in health infrastructure 4.6. Strengthen the capacity of health workers

COVID-19, coronavirus disease 2019.

#### Theme 1: Approaches and opportunities for successful COVID-19 response, care and management

This theme outlines the ministry’s diverse strategies for pandemic response, both in the community and at health facilities, aimed at promoting service utilisation by the public.

**Code 1.1: Well-coordinated COVID-19 response:** A well-organised and coordinated response was adopted under the guidance of the national task force, overseen by the prime minister’s office at various levels of government. This decentralised approach involved the deployment of district task forces (DTFs) and focal persons down to the village level. These individuals were entrusted with responsibilities such as policy formulation and resource mobilisation:

‘We had a unified front of the pandemic response that was coordinated and the office of prime minister with the chair of the National task force as the president of the country, but all arms in the government were involved in the pandemic response, either the level of advocacy or resource mobilization or health care service delivery. As the Ministry of Health, we coordinated the response on behalf of the national coordination, that was coordinated under the national task force.’ (KII, MoH)

**Code 1.2: Health facility response:** Health structures and some government structures were repurposed to accommodate COVID-19 patients as case management, isolation centres or intensive care units, and health workers were trained to respond to COVID-19 emergencies:

‘When it comes to the second wave, we also saw patients who were very sick and needed to be on oxygen. So, in other words Namboole which was initially a non-traditional isolation facility, later actually became a treatment center, like a hospital for severe COVID-19.’ (KI, MoH)

**Code 1.3: Integration:** Respondents found minimal integration of COVID-19 with routine care, particularly in the pandemic’s early stages. Focus was skewed towards tuberculosis (TB), human immunodeficiency virus (HIV) and antenatal services, neglecting other health conditions:

‘I think the integration was not much, there was very little integration but what I remember is that like TB detection and COVID, we tried to integrate those two because when you have TB you cough, you have flu, you have chest pain, the same thing, so it’s better to do what [*integrate*]. So, we tried, integration, but in other sectors, other programs, not very much.’ (KII, MoH)

*Community delivery and utilisation*: Health service delivery was primarily through home-based care, utilising VHT structures to assess COVID-19 cases and provide treatment for individuals with non-communicable diseases or requiring routine care, such as people living with HIV (PLHA), diabetic and hypertensive patients:

‘Other departments within IDI for example the clinic that provide HIV services, they had to figure out ways of distributing drugs in communities rather than people coming to health facilities.’ (KI, CDC)

**Code 1.4: Communication strategy:** *Risk communication and digitisation (Code 1.5)*: The MoH centrally managed risk communication, primarily using mass media such as television and radio, supplemented by outreach campaigns and distribution of informational materials. To minimise patient-health worker exposure, digital services were employed, including telephonic screening for COVID-19 through a toll-free line. Treatment was prescribed for pickup at nearby pharmacies, with follow-ups conducted telephonically to enhance communication between health workers and the community:

‘The minister … she used to hold media briefs, both at the radio and at the media center, so that really also helped a lot. That’s why the message really sank …’ (KI, MoH)

**Code 1.6: Building on existing capacity and experience:** Respondents drew on past experiences managing similar outbreaks, highlighting it as the major strength in responding to the pandemic. The presence of established systems and structures, including surveillance and human resources, played a crucial role in successfully implementing their response efforts:

‘Uganda is a country that is every now and then responding to an outbreak of some condition, be it Ebola, yellow fever, sometimes local, sometimes imported and so there was some capacity in the country, for outbreak response.’ (KI, MoH)

#### Theme 2: Negative effects of the COVID-19 pandemic on health service delivery

**Code 2.1: Disruption of routine services:** Despite health system modifications involving infrastructural repurposing, the development of isolation and intensive care centres, and the enhancement of medical emergency services alongside capacity building for health workers, respondents found massive disruption of routine health services. The repurposing of structures led to space shortages and worsened the human resource gap in some health units, with health workers redirected to the COVID-19 response. There was a biased focus on COVID-19 services at the expense of other health services, as resources were redirected from various programmes to support COVID-19 management. This shift is attributed to the loss of lives from ailments other than COVID-19, compromising the quality of care:

‘We took money from maternal and child health programming and put it into COVID. We took staff from those offices and made them sit on pillars and made them pivot their activities to support the COVID response, including the school-based surveillance staff and the OVC. A lot of assets or partners were leveraged to deliver COVID services too and that was a strain on everybody.’ (KI, USAID)

Imposing lockdown also hindered health workers from accessing health facilities to serve patients and hindered patients from accessing health services because of transport restrictions. There was also general fear by both patients and health workers to go to hospitals because of fear of contracting COVID-19 while in hospital, further disrupting health services:

‘The utilization of the health services almost went down. First, there was a problem of people reaching the health facilities, there was a problem of movement. So, you would find that people were told to stay where they were, so accessing the health services was a problem.’ (KI, MoH)

**Code 2.2: Pressure on health resources including an increased workload:** There was a shortage of crucial medical resources, such as oxygen, bed space and healthcare personnel because of an increase in COVID-19 cases. Despite efforts to repurpose facilities and train health workers for pandemic response, these challenges severely compromised the health system’s ability to provide effective services:

‘Really, the health system was overwhelmed, and we were unable to give services that we should have in terms of oxygen, in terms of human resource, in terms of even space and infrastructures.’ (KI, Mental Health-partner)

#### Theme 3: Barriers to implementation of COVID-19 response strategies

**Code 3.1: Government mistrust:** Government mistrust arose from miscommunication, false information and uncertainties about the disease’s existence. Some viewed lockdowns as a punitive measure and others believed that the government invented them for financial gain. Doubts persisted as COVID-19 disease did not appear to affect their communities, creating challenges in implementing prevention programmes:

‘At some point, people thought the government just wanted to keep them in their houses, they wanted what to eat because they thought that what they call lockdown was a punishment.’ (KII, MoH)

‘Well of course, the entire COVID was taken by mixed feelings at some point the general population thought that it was overreacting, sometimes they even doubted the presence of COVID in the country and you see once people have those thoughts it affects how they react or view the health system, so there was some ingrown miss trust of the health systems.’ (KII, Mental Health-partner)

**Code 3.2: Dynamic nature of the pandemic:** Key informants noticed the dynamic nature of the pandemic, marked by sudden spikes in cases and supply chain gaps. There was continuous planning and adaptations as their response plans became obsolete over time because of the evolving pandemic:

‘Sometimes what we planned for manifested, but again, the COVID-19 situation kept on changing. So, we would plan for this, and tomorrow they are changing, and you have to go back to the drawing board.’ (KI, Mental Health-partner)

**Code 3.3: Violation of protocols:** Mass violations of established protocols and guidelines were cited as undermining pandemic response efforts. Economic survival drove individuals to seek work, leading to non-compliance with COVID-19 prevention protocols:

‘In a situation where pretty much, nobody trusts their government anywhere on this globe, that weak public trust in government everywhere made it hard to implement classic public health measures. But people did the best they could, given their concerns and their lack of willingness to comply, and just sort of struggling with these realities …’ (KI, USAID)

**Code 3.4: Inadequate funds:** Limited funds and resources for the pandemic response were emphasised, exacerbated by rising prices of essential commodities, including masks, which were required as a medical countermeasure, because of global factory closures and high demand. The absence of a designated budget for emergencies further compounded the issue, underscored by the misuse of funds by certain officials:

‘The data required to respond to this outbreak also was a big issue. It was a global demand for PPEs for example, for medicine, for vaccines and that increased the price and of course as for the third world it wasn’t and the number of donor funding or contributions was not enough to support all those demands that, I mean, the price that was so high. So, there was increased price and yet the cases were many.’ (KII, CDC)

#### Theme 4: Suggestions for future preparedness during pandemics

**Code 4.1: Focus on continuity of health services amid pandemics:** The continuity pillar was established to plan for and implement essential health services. This involved conducting meetings, drafting policy guidelines and forming an incident management team to reactivate neglected health services. Activities included enhancing ambulance access for emergencies, extending prescription durations for long-term ailments such as Anti Retro Viral drugs, and providing health workers and supplies to support other health departments:

‘WHO initiated what they call continuity of essential health services, we used to make sure that whatever we were doing, does not impact negatively on routine service.’ (KI, MoH)

**Code 4.2: To improve public awareness:** Respondents emphasised increasing awareness of the pandemic by enhancing public perception of danger through risk communication and demystifying through enormous community engagement approaches such as outreaches and media to address misconceptions about COVID-19 that caused doubt and government mistrust:

‘The most important thing is public sensitization and awareness. So that they know and they take responsibility for their health. So, what the government is doing is additional, not to respond to the threats. But if the public is not aware, the public is not responsive; it becomes difficult when they start resisting.’ (KII, MoH)

**Code 4.3: To improve surveillance:** They also strongly recommended improved surveillance systems that readily detect new diseases and cases to enable rapid response:

‘I think we have a responsibility to detect them and notify and mount an effective response quickly. All of this has to be done in a period of days if there is a chance of stopping a future outbreak.’ (KI, CDC)

**Code 4.4: To improve coordination and leadership:** Better coordination and leadership coupled with preparedness for pandemics before they occur in terms of resource planning and reservation was encouraged to cater to other essential services besides a pandemic:

‘The first strategy is to have coordinated leadership, as we did through having a strategic team and the incident management team in the different pillars. We sit and plan for this pandemic because everyone has expertise in different areas, and then it’s coordinated. Each pillar comes in with its motive and coordinated, and then the leadership takes in.’ (KI, MoH)

**Code 4.5: Investment in health infrastructure:** Respondents emphasised the importance of establishing additional healthcare infrastructure, such as expanding hospitals and ensuring they are equipped with essential medical supplies such as oxygen, to better prepare for future pandemics to minimise disruption of other programmes:

‘Let the pandemics find us prepared. So, Let’s have in place areas prepared for handling pandemics, for handling patients who catch the disease. Let’s have in place funds because we saw that funds were only being solicited for, but we should have like an emergency fund that can be utilized when such pandemics come up, and then, we should have a reserve health workforce just like you see a reserve army in the security forces and then I think with those we can be able to handle future pandemics.’ (KI, MoH)

**Code 4.6: Strengthen the capacity of health workers:** They advocated for enhancing the capacity of health workers, including community health workers to readily respond to cases at the community level. Training in areas such as infection prevention, identifying asymptomatic cases and managing the psycho-social well-being of patients and health workers were recommended:

‘I think they should do drills often. Some healthcare workers never get trained on some of these epidemics. Once it comes, it takes them unaware and they don’t know how to – they do not have the confidence. So, I think those skills are really needed. Also, the healthcare workers need to have communication skills because we noted that there was a lot of distress in taking care of patients with COVID-19 because there were gaps in communication, how to talk to someone who is in distress, … and even how to communicate to the community because we encourage them to deal with, and demystify the myths around COVID-19. But also, we need to give health workers skills on how to take care of their own well-being during stress.’ (KI, Mental Health-partner)

‘a more skilled community level workforce hm should be invested in, so that this can serve as good as the Village Health Teams … I think there are a number of strains that have moved on to the health system within the health facility that could have been managed at the community level.’ (KI, WHO)

## Discussion

Our findings revealed that identifying COVID-19 cases triggered substantial disruptions in routine health services and erosion of trust in government actions. The pandemic imposed an increased workload on health workers, adding to the negative impacts experienced. The challenges encountered while preventing and containing COVID-19 were multifaceted: encompassing the dynamic nature of the pandemic, financial constraints and instances of violation of COVID-19 prevention protocols. Despite these encounters, the ministry’s prior experience in managing pandemics and the presence of established structures emerged as opportunities for the successful implementation of the COVID-19 response.

The disruption of health services directly resulted from restricted access to healthcare facilities imposed by the lockdown. The overwhelming emphasis on COVID-19 management during this period left other critical disease response efforts neglected. This observation aligns with prior studies’ findings, reinforcing that the pandemic’s impact extends beyond COVID-19 itself.^9,15,18^ Respondents underscore the need for concerted efforts to restore these essential health services, a sentiment reflected in introducing a ‘continuity of health service’ pillar. Notably, this initiative appears to have been implemented after a comprehensive assessment of the adverse effects stemming from a disproportionate focus on COVID-19 services at the expense of other health priorities. Recognising these negative implications has prompted a strategic shift, integrating COVID-19 management into routine healthcare programmes. This approach acknowledges the interconnectedness of various health services. It aims to rectify the initial bias, ensuring a more comprehensive and inclusive healthcare response in the ongoing battle against the pandemic, as further emphasised by respondents.^19^

The study revealed that healthcare workers experienced significant fear of going to hospitals because of the risk of contracting COVID-19, which further disrupted the delivery of essential health services. Studies associate related anxiety with an impact on a substantial toll on their mental well-being. The constant apprehension about potential exposure to the virus, combined with concerns about transmitting it to their families, could also contribute to heightened stress, burnout and emotional strain.^20^ In addition, the pressure to adhere to stringent infection control measures, coupled with the emotional burden of witnessing severe illness and death, could have led to feelings of isolation and diminished job satisfaction. These challenges likely contributed to increased absenteeism and lower morale among healthcare workers.^21,22^ Furthermore, other studies highlight social isolation fears and economic psychological distress among community members because of financial insecurities from the lockdown measures.^23^ In the light of these findings, respondents recommended that training for healthcare workers should incorporate strategies for building mental resilience, to better equip them to cope with the emotional and psychological demands of such high-risk environments. This has similarly been observed and recommended in previous studies.^24^

The pervasive lack of information within the community emerged recurrently, influencing various aspects of the pandemic’s early stages. This posed a significant obstacle to the effective implementation of management and prevention interventions for COVID-19, as well as the promotion of vaccine acceptance. Despite proactive efforts by the response team to disseminate comprehensive information on risks, prevention, management and vaccines, the persistent lack of awareness regarding different facets of COVID-19 remains a prominent issue even for similar contexts.^25,26^ This knowledge gap is notably implicated in fostering government mistrust, perpetuating myths and misinformation and contributing to a general reluctance to adhere to COVID-19 protocols and the COVID-19 vaccine. Despite endeavours to bridge these informational divides, failure to address knowledge gaps and dispel associated myths and misconceptions persists in the current state of the pandemic.^27^ This ongoing deficiency in understanding not only influences low-risk perception but also poses a threat to acceptance and compliance with future response efforts for the pandemic.^28,29^ Recognising and actively addressing these informational gaps is imperative for fostering informed decision-making, instilling public confidence and ultimately enhancing the success of COVID-19 prevention efforts as well as possible future pandemics.^30^

The experience gained from managing previous viral outbreaks, such as Ebola and Marburg viruses, has been acknowledged as a valuable asset in confronting the challenges posed by the COVID-19 pandemic. Respondents have pointed to Uganda’s success in leading responses to Ebola outbreaks in West Africa and leveraging lessons learned from past encounters with similar pathogens. Undoubtedly, this accumulated expertise offers a foundation for effective implementation of COVID-19 programmes.^31^

However, the pandemic has shed light on critical shortcomings in global health infrastructure and response mechanisms despite the purported advantages of prior experience. For instance, the repurposing of existing health facilities for COVID-19 cases revealed a lack of preparedness in terms of infrastructure, particularly in creating sufficient isolation units. Logistical issues further compounded the situation, with shortages in essential supplies such as personal protective equipment (PPE) and oxygen, necessary for safeguarding healthcare workers and treating patients.

A notable strength of this study is that the findings document the perspectives of key informants on the cascade and effects of COVID-19 on healthcare programmes, sharing lessons learned during the pandemic and how challenges were addressed.^15^ We acknowledge limitations in recall bias, which limits phenomenological documentation of response. We, however, minimised this by referring to documentation of the COVID-19 response by the Uganda MoH.^2^

## Conclusion

The COVID-19 epidemic not only had negative effects on the healthcare system but also provided opportunities for further improvement of healthcare delivery in the country. The epidemic disrupted health programmes, severely affecting access to healthcare in communities. The dedicated departments at the MoH and other ministries responsible for epidemic risk determination and response in the country need to be strengthened.
